# Older Adults’ Views about Blood Biomarker Testing for Alzheimer’s Disease: Update from the U.S. National Poll on Healthy Aging

**DOI:** 10.1002/alz.089261

**Published:** 2025-01-09

**Authors:** Chelsea G Cox, J. Scott Roberts

**Affiliations:** ^1^ University of Michigan School of Public Health, Ann Arbor, MI USA

## Abstract

**Background:**

Blood‐based biomarker testing (BBT) for Alzheimer’s disease (AD) to detect amyloid‐beta and tau is becoming part of dementia care, with likely future clinical use in asymptomatic populations. Little is known about older adults’ awareness of BBT or their perceptions of its potential benefits, risks, and limitations.

**Method:**

We analyzed data from the March 2023 fielding of the University of Michigan National Poll on Healthy Aging, a nationally representative web and telephone survey of community‐dwelling older U.S. adults. Poll respondents ages 65‐80 were provided a brief definition of BBT for AD and asked about their 1) awareness of BBT, 2) perceptions of its risks and benefits, and 3) anticipated responses if they tested positive. Multivariable logistic regression analyses examined self‐reported sociodemographic (i.e., age, sex, race and ethnicity, education, income, employment, martial status) and clinical characteristics (i.e., physical health, mental health, subjective memory, family history of dementia) associated with BBT awareness and anticipated negative responses to a positive AD blood biomarker result.

**Result:**

Of 1,298 U.S. adults surveyed, 19% said they were at least somewhat familiar with BBT for AD. Older adults with more education and better mental health had higher odds of BBT awareness (p<0.05). Overall, older adults were more likely to endorse possible benefits versus risks of testing. For example, 76% said BBT could be useful to inform medical care or advance planning, while 32% endorsed concern about the privacy of their results. More than half of older adults were somewhat or very likely to say that a positive BBT result would prompt potential negative reactions, including beliefs that they would develop AD (72%), have significant distress (61%), or be viewed differently by others (54%). Being female, non‐Hispanic White (compared to Hispanic), and having a family history of dementia were associated with higher odds of anticipating negative outcomes of BBT (p<0.05).

**Conclusion:**

Most older U.S. adults are not aware of BBT for AD but view it as potentially beneficial for informing clinical care. Many older adults anticipate potential social and psychological challenges of receiving a positive BBT result, with these test perceptions differing significantly by gender and race/ethnicity.

